# Activation and induction of antigen-specific T follicular helper cells play a critical role in recombinant SARS-CoV-2 RBD vaccine-induced humoral responses

**DOI:** 10.1186/s43556-023-00145-z

**Published:** 2023-10-19

**Authors:** Songhao Yang, Liangwei Duan, Chan Wang, Cuiying Zhang, Siyu Hou, Hao Wang, Jiahui Song, Tingting Zhang, Zihua Li, Mingxia Wang, Jing Tang, Qianqian Zheng, Hui Wang, Qi Wang, Wei Zhao

**Affiliations:** 1 School of Basic Medical Science, Ningxia Medical University, Yinchuan, Ningxia Hui Autonomous Region 750004 People’s Republic of China; 2 Key Laboratory of Hydatid Disease, Ningxia Medical University, Yinchuan, Ningxia Hui Autonomous Region 750004 People’s Republic of China; 3 Center of Scientific Technology, Ningxia Medical University, Yinchuan, Ningxia Hui Autonomous Region 750004 People’s Republic of China; 4 Henan Key Laboratory of Immunology and Targeted Drugs, School of Laboratory Medicine, Xinxiang Medical University, Xinxiang, 453003 Henan Province China; 5 Henan Collaborative Innovation Center of Molecular Diagnosis and Laboratory Medicine, Xinxiang Medical University, Xinxiang, 453003 Henan Province China

**Keywords:** SARS-CoV-2, RBD protein, Tfh cells, B cells, Humoral response

## Abstract

The role of follicular T helper (Tfh) cells in humoral response has been considered essential in recent years. Understanding how Tfh cells control complex humoral immunity is critical to developing strategies to improve the efficacy of vaccines against SARS-CoV-2 and other emerging pathogens. However, the immunologic mechanism of Tfh cells in SARS-CoV-2 receptor binding domain (RBD) vaccine strategy is limited. In this study, we expressed and purified recombinant SARS-CoV-2 RBD protein in *Drosophila* S2 cells for the first time and explored the mechanism of Tfh cells induced by RBD vaccine in humoral immune response. We mapped the dynamic of Tfh cell in lymph node and spleen following RBD vaccination and revealed the relationship between Tfh cells and humoral immune response induced by SARS-CoV-2 RBD vaccine through correlation analysis, blocking of IL-21 signaling pathway, and co-culture of Tfh with memory B cells. Recombinant RBD protein elicited a predominant Tfh1 and Tfh1-17 subset response and strong GC responses in spleen and lymph nodes, especially to enhanced vaccination. IL-21 secreted by Tfh cells affected the development and differentiation of B cells and played a key role in the humoral immune response. These observations will help us further understand the mechanism of protective immune response induced by COVID-19 vaccine and has guiding significance for the development of vaccines against newly emerging mutants.

**Supplementary Information:**

The online version contains supplementary material available at 10.1186/s43556-023-00145-z.

## Highlights

**•** Recombinant SARS-CoV-2 RBD protein from *Drosophila* S2 cells fostered functional Tfh cells and GC responses.

• Tfh cells modulated the SARS-CoV-2 RBD protein-induced humoral immunities through the IL-21/IL-21R signaling pathway and efficiently induced the differentiation of memory B cells via IL-21.

**Supplementary Information:**

The online version contains supplementary material available at 10.1186/s43556-023-00145-z.

## Introduction

The outbreak of COVID-19 in 2019 has posed a huge threat to human health. The emergence of the Omicron variant and its offspring subvariants, such as BQ.1.1 and XBB, has encouraged SARS-CoV-2 to continue to rage around the world [1, 2]. Studies have shown that XBB increases the risk of reinfection than other prevalent sub-lineages of the Omicron lineage [3]. The rapid rise of these subvariants and their extensive spike mutations have raised concerns that they could further impair the efficacy of current COVID-19 vaccine and monoclonal antibody therapies. Therefore, it is essential to develop more effective vaccines against various variants.

An effective vaccine can prevent and control infectious diseases through inducing high-affinity antibodies and memory B cells (MBC) in germinal centers (GC) of secondary lymphoid organs. The formation of GC relies on follicular T helper (Tfh) cells, which facilitates the transformation of GC B cells into longevous plasma cells (PC) and MBC by secreting costimulatory molecules and cytokines [4]. In recent years, the importance of Tfh cell in the humoral immunity is becoming clear [5]. A report showed Tfh with ICOS production correlated with antibody after vaccination with hepatitis B vaccine [5, 6]. Research on influenza vaccine showed Tfh cells efficiently helped antibodies production and MBC differentiation [6, 7]. Growing evidence has shown a key role for Tfh cells in vaccine-induced immunity, and understanding how Tfh cells control complex humoral immunity is critical to developing strategies to improve the efficacy of vaccines against SARS-CoV-2 and its emerging variants [8, 9]. Several recent studies reported that COVID-19 mRNA vaccines fostered antigen-specific Tfh cells to engender robust neutralizing antibody responses [4, 10]. *Cavazzoni *et al. [11] proved that reduction of Tfh cells led to decreased GC responses and antigen-specific IgG secretion through Tfh cell-deletion (Tfh-DTR) mice after SARS-CoV-2 spike protein vaccination. Overall, these data emphasize that Tfh cells are beneficial to control SARS-CoV-2 infection by conferring long-term immune protection.

Tfh cells, as a crucial subset of CD4^+^T cells, are characterized by expressing high levels of CXCR5, PD-1, ICOS, IL-21, and Bcl-6. Bcl6 is required for early CXCR5 expression and plays a positive regulatory role in Tfh differentiation and development [12, 13]. IL-21 is not only required for Tfh cell differentiation, but IL-21 is also an effective inducer for the production of high-affinity antibodies and PC differentiation [12, 14]. The function of Tfh cells in immunity following vaccination is an emerging field of research, and these cells primarily associate with disease progression and pathology. It is now increasingly recognized that Tfh cells play a key role in mediating protection against a wide range of pathogens [15].

Currently, it has been found that the SARS-CoV-2 receptor binding domain (RBD) subunit vaccine has been found could facilitate adapted immunity against virus infection [16–18]. RBD protein, which serves as an essential role in virus attachment and a major target for host immune responses, plays an important role in recognizing angiotensin-converting enzyme 2 (ACE2) and contributing to fusion and viral entry into the host cell [19–22]. Therefore, RBD protein has been an ideal antigen for numerous vaccine candidates. *Coria *et al. [23]showed RBD-based COVID-19 vaccine increased neutralizing antibodies, specific GC B cells and conferred protection against SARS-CoV-2 infection. Additionally, *Cong* et al. [24] proved 3-dose of RBD vaccine was sufficient to elicit Tfh cell responses. Nevertheless, there are a lack of in-depth studies to explore the immunological mechanism of their effects in Tfh-mediated immunity following the SARS-CoV-2 RBD vaccine immunization.

Here, we investigated the dynamic change and function of Tfh cells by recombinant SARS-CoV-2 RBD protein expressed in *Drosophila* S2 cells and the immunologic mechanism in humoral responses after prime and booster vaccination, and presented that the Tfh cell response in spleen and lymph node correlated with B cell response and provided help to MBC.

## Results

### Design and development of a recombinant RBD subunit vaccine antigen

**Fig. 1:**
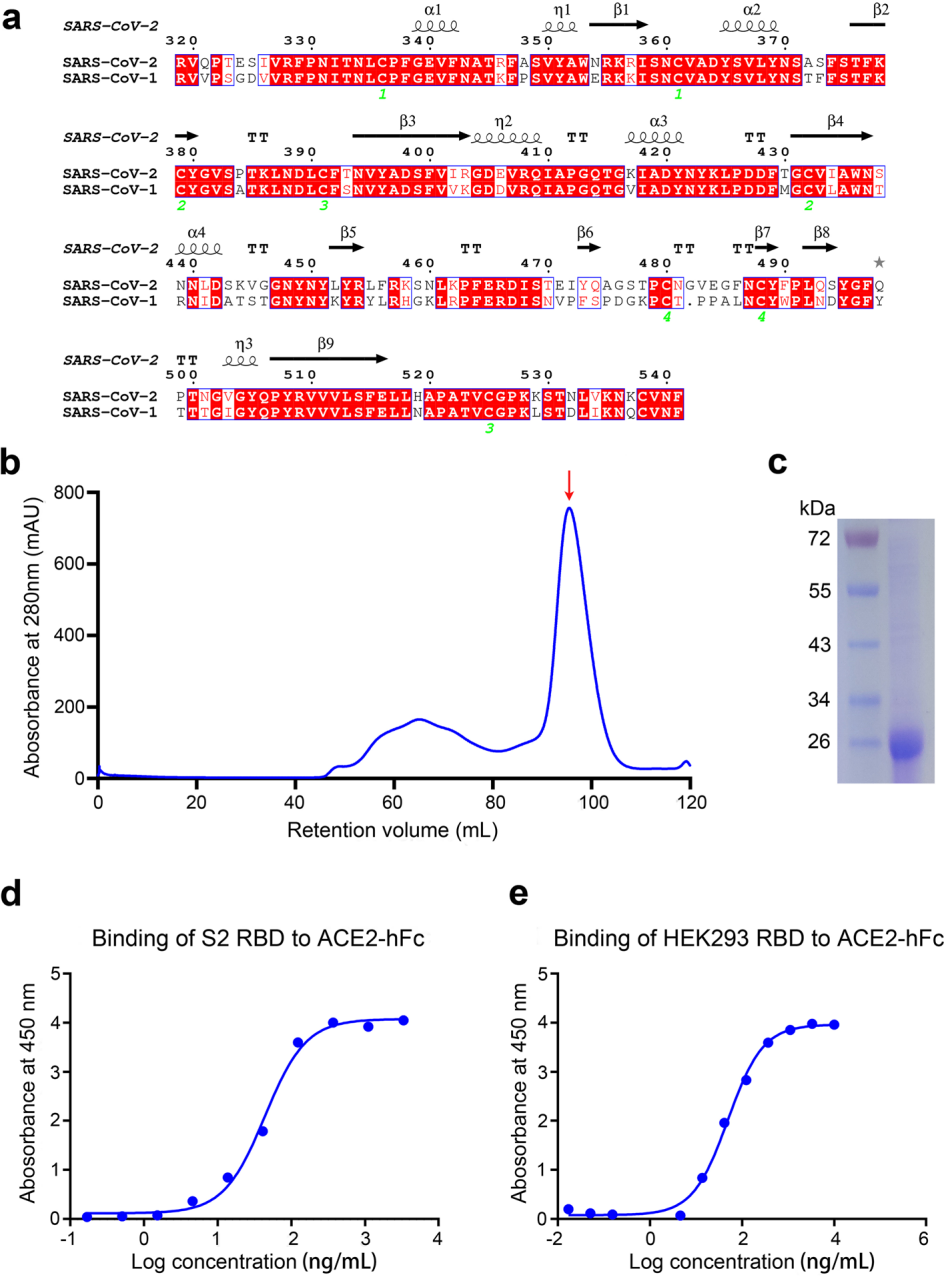
Design and development of a recombinant RBD subunit vaccine antigen. **a** Sequence alignment of SARS-CoV-1 RBD (residues 306–527) and SARS-CoV-2 RBD (residues 319–541). The secondary structures of SARS-CoV-2 RBD are shown above the alignment. The red highlight indicates conserved residues. The green number below the alignment represents the conserved cysteines. **b** Size exclusion chromatography profile of RBD protein with predominantly monomeric peak at ∼95.9 mL on a HiLoad 16/600 Superdex 200 column. The proposed peak RBD protein is indicated with a red arrow. **c** SDS-PAGE analysis of SARS-CoV-2 RBD at reduced conditions. Protein molecular weight marker (kDa) are indicated on the left. **d**, **e** Binding curve of purified recombinant S2 RBD protein (**d**) and marketed HEK293 RBD protein **(e)** to ACE2-hFc measured by ELISA

RBD protein acts as the main area of action for neutralizing antibodies induced during virus infection or following vaccination [21, 22], which play a vital role in protecting severe COVID-19 and infection. Therefore, it is a component of almost all vaccine antigens. The RBD of SARS-CoV-2 shares 74% amino acid sequence identity with its equivalent from SARS-CoV-1 (Fig. 1a). By sequence alignment and secondary structures of SARS-CoV-2 and SARS-CoV-1 RBDs, we identified the region of SARS-CoV-2 RBD used in this study at residues 331 to 529 of the spike protein (Fig. 1a). The resultant RBD is different from SARS-CoV-1 RBD (residues 318 to 510) and the most common SARS-CoV-2 RBD (residues 319 to 541) version [21], in order to avoid flexible termini as well as an unpaired cysteine, Cys391, which forms an interchain disulfide bond with Cys525.

Elution of individual spikes in the size exclusion column indicated that the purified RBD protein has high purity and homogeneity as a monomer (Fig. 1b). In addition, we detected the purity of the proteins by reducing sodium lauryl sulfate–polyacrylamide gel electrophoresis (SDS-PAGE) analysis (Fig. 1c). Notably, the expression of RBD protein in S2 cells is very high with a purified yield of ~ 100 mg/L culture. As expected, purified RBD protein bound strongly to the ACE2 receptor with a half-maximal effective concentration (EC50) value of 43.84 ng/mL (Fig. 1), which was slightly lower than that observed for a commercialized RBD protein purified from HEK293 cells (EC_50_ = 49.33 ng/mL) (Fig. 1d, e). This indicates RBD protein produced in S2 cells has a slightly higher affinity for ACE2 than RBD protein produced in HEK293 cells, suggesting its potential to be a good immunogen. Overall, these results indicate that a recombinant RBD subunit vaccine antigen were successfully obtained.

### Induced ICOS^+^CXCR5^+^CD4^+^T cells recognize SARS-CoV-2 RBD antigen at early stage after vaccination

**Fig. 2:**
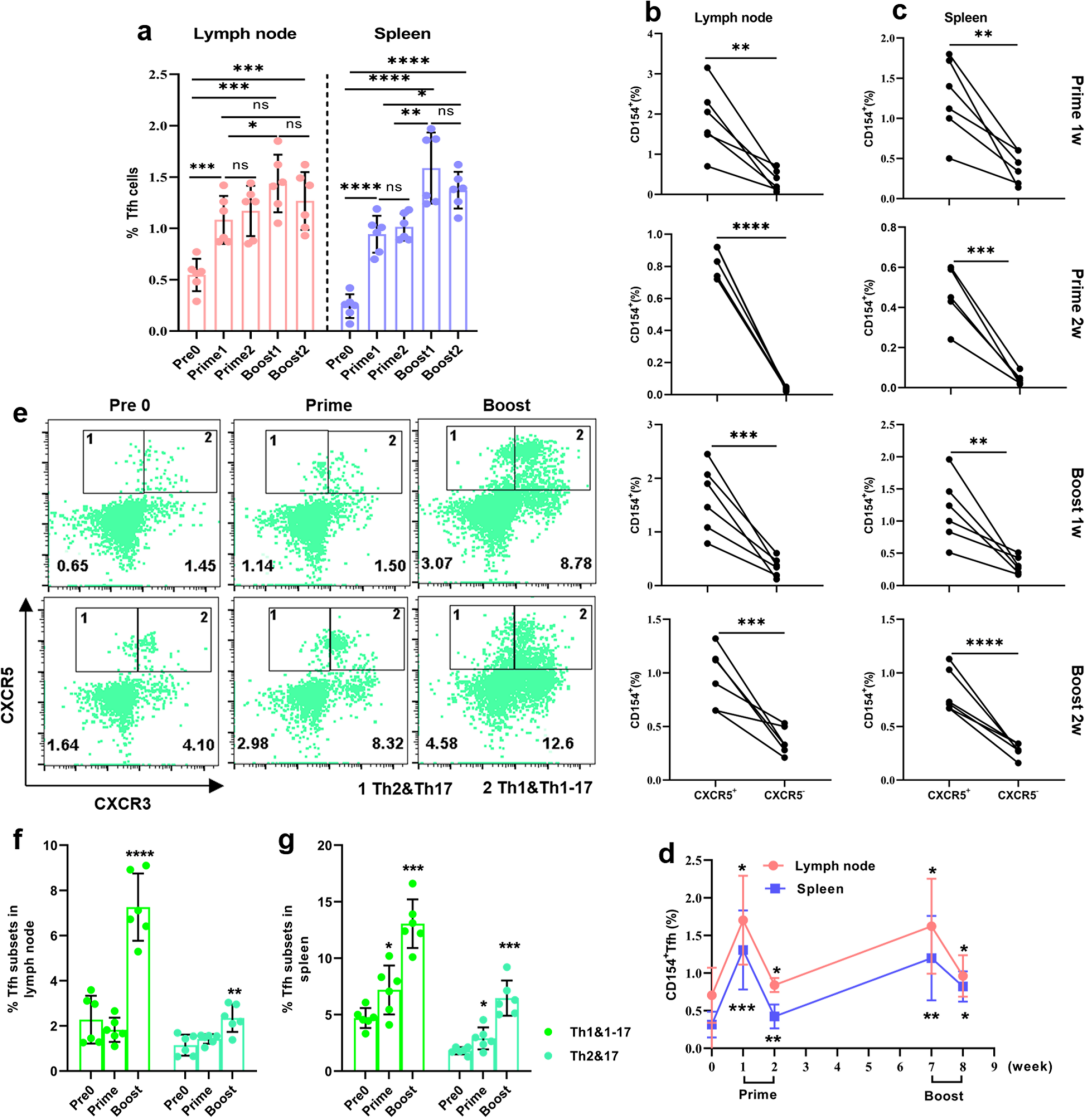
Induced Tfh cells recognized SARS-CoV-2 RBD antigen and triggered a predominant Tfh1 and Tfh1-17 subset response. **a** The dynamic changes of Tfh cells in lymph node and spleen. **b**, **c** The frequencies of CD154 in CXCR5^+^ and CXCR5^−^ cells in lymph node (**b**) and spleen (**c**) after prime and booster vaccination. **d** Statistical analysis showing the frequency of CD154 in CXCR5^+^ cells from lymph node and spleen. **e** Representative flow cytometry plots showing CXCR3^+^ and CXCR3^−^ subsets. 1, CXCR3^−^ (Tfh2 and Tfh17) gate; 2, CXCR3^+^ (Tfh1 and Tfh1-17) gate. **f** Statistical analysis showing the frequencies of CXCR3^+^ and CXCR3.^−^ subsets. One way ANOVA test, n = 5–6. *: *P* < 0.05, **: *P* < 0.01, ***:* P* < 0.001, ****:* P* < 0.0001

The initiation and maintenance of humoral immunity depend on Tfh cells. Therefore, we detected the dynamic change of Tfh cells in spleen and lymph node after prime and booster vaccination. We characterized Tfh cells as CD4^+^B220^−^CD44^+^CXCR5^+^ICOS^+^ in T cells by flow cytometry (Fig. S1) [7]. A prominent increase in Tfh cells in lymph node were observed after the prime and enhanced immunization (*p* < 0.001, *p* = 0.01, Fig. 2a). There was a similar pattern in spleen after immunization (*p* < 0.0001, *p* = 0.002, Fig. 2a).

Next, we stimulated lymphocyte from spleen and lymph node at day 7 and 14 after prime and booster vaccination for 6 h with RBD protein in the presence of brefeldin A to verify the responses of Tfh cells to RBD proteins. CD154 is well known as a common functional marker of antigen-activated T cells, we therefore analyzed the expression of CD154 in Tfh cells (CXCR5^+^ICOS^+^) and non-Tfh cells (CXCR5^−^ICOS^+^) from spleen and lymph node after prime and booster vaccination. The high expression of CD154 was mainly occurred in CXCR5^+^ICOS^+^ cells from spleen and lymph node after immunization (*p* = 0.002, *p* < 0.001, *p* < 0.0001, Fig. 2b, c), and was significantly increased at day 7 (*p* = 0.02, *p* < 0.001, *p* = 0.001, Fig. 2b, c), and decreased at day 14 after prime and booster vaccination (*p* = 0.02, *p* = 0.001). In addition, the overall level of CD154 expression in CXCR5^+^ICOS^+^ cells from lymph node remained significantly higher than that from spleen (Fig. 2d). In general, recombinant RBD protein induced CXCR5^+^ICOS^+^ Tfh cells contained antigen-specific cells.

### RBD protein triggered a predominant Tfh1 and Tfh1-17 subsets response following booster vaccination

To determine the dynamic kinetics of Tfh cell subpopulations in spleen and lymph node following immunization with RBD protein, we used CXCR5 and CXCR3 chemokine receptor markers to characterize Tfh subsets including CXCR3^+^ (Tfh1 and Tfh1-17), and CXCR3^−^ (Tfh2 and Tfh17). We found that there was no significant difference in lymph node was observed after prime vaccination (Fig. 2e, f). However, a modest increase of CXCR3^+^ and CXCR3^−^ subsets in spleen was observed after prime vaccination (*p* = 0.03, Fig. 2e, g). Notably, booster vaccination led to a robust increase in CXCR3^+^ subset in spleen and lymph node (*p* < 0.0001, *p* < 0.001, Fig. 2f, g). We also saw modestly increased frequency of CXCR3^−^ subset in spleen and lymph node (*p* = 0.006, *p* < 0.001). The response of CXCR3^+^ subset was stronger than CXCR3^−^ subset. In summary, these results demonstrated that RBD protein mainly skewed the distribution of Tfh subsets toward Tfh1 and Tfh1-17 response following booster vaccination.

### Elicitation of functional Tfh cell response by regulating the expression of factors associated with growth and development

**Fig. 3:**
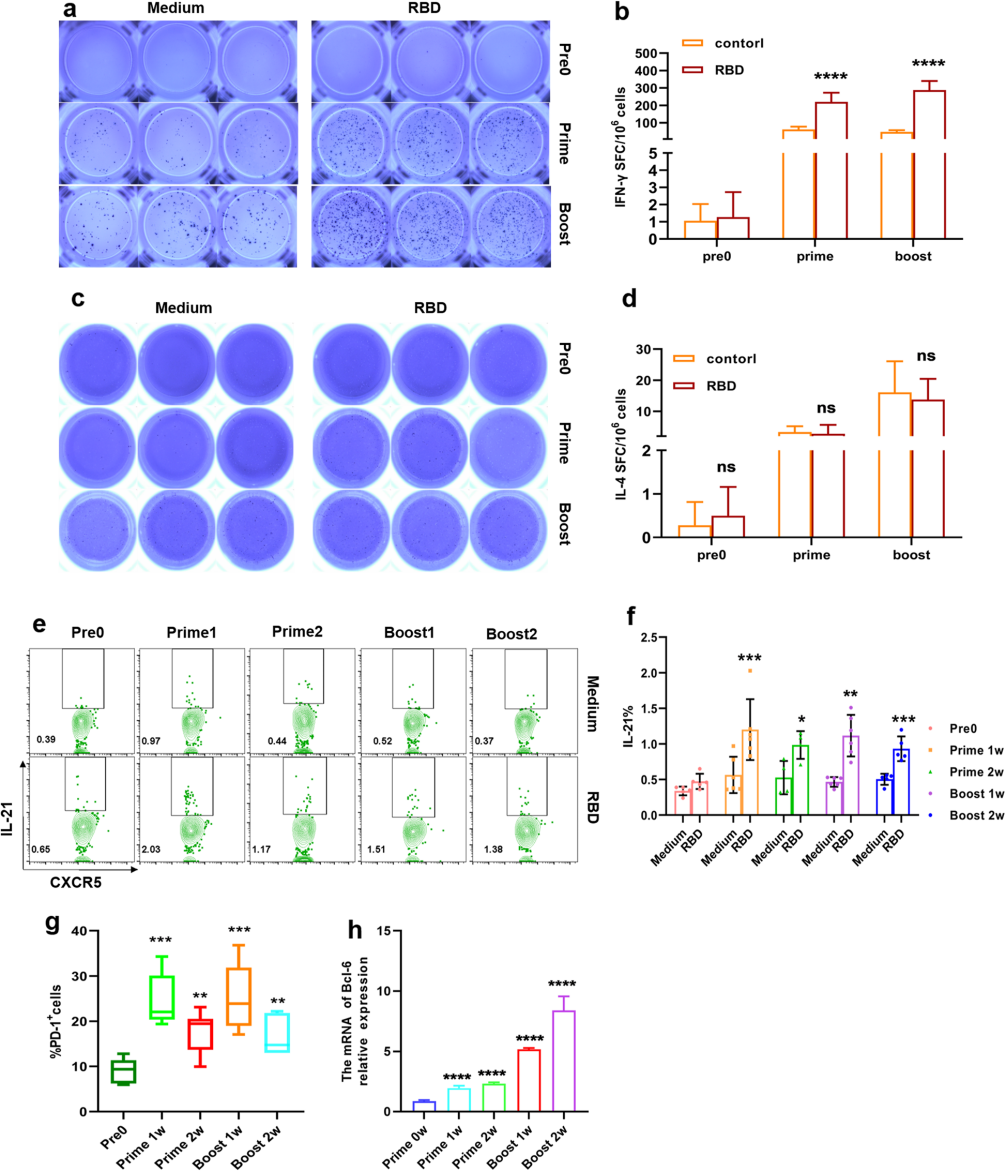
Elicitation of functional Tfh cell response by regulating the expression of factors associated with growth and development. **a**, **c** Representative IFN-γ and IL-4 ELISpot images were shown. **b**, **d** Comparative IFN-γ and IL-4 ELISpot spot forming units (SFUs) per 1 × 10^6^ lymphocytes in spleen after vaccination. **e** Representative flow cytometry plots showing IL-21 in splenic lymphocytes following prime and booster vaccination. **f**, **g** Statistical analysis of the expression of IL-21 (**f**) and PD-1 g. **h** qRT-PCR detected the Bcl-6 mRNA expression in splenic lymphocytes stimulated with RBD protein after vaccination. two-tailed Student’s t-test (**b**, **d**, **f**) and One way ANOVA test (**g**, **h**), n = 5–6. *: *P* < 0.05, **: *P* < 0.01, ***:* P* < 0.001, ****:* P* < 0.0001, ns: no significance

We first examined whether RBD immunization induced functional T cell response. ELISpot results showed that the number of IFN-γ-T cells was significantly increased after immunization, but booster immunization did not improve the number of IFN-γ-T cells (*p* < 0.0001, Fig. 3a, b). However, we did not detect differential expression of IL-4, its overall level increased with increasing immunizations (Fig. 3c, d).

To further investigate the immune mechanism of Tfh cell response regulated by RBD proteins, we evaluated the expression of transcription factors and key molecules associated with Tfh cell growth, development and function. The levels of IL-21and PD-1 were remarkable increase after immunization (*p* = 0.0003, *p* = 0.02, *p* = 0.005, *p* < 0.0001, *p* = 0.003, Fig. 3e-g). We observed an around twofold higher level of Bcl-6 production after prime vaccination and more than fivefold higher level after booster vaccination (*p* < 0.0001, Fig. 3h). Together, these data highlighted that RBD protein elicited functional Tfh cell response by regulating the expression of factors associated with growth and development.

### RBD protein-induced Tfh cell responses correlated with B cell responses

**Fig. 4:**
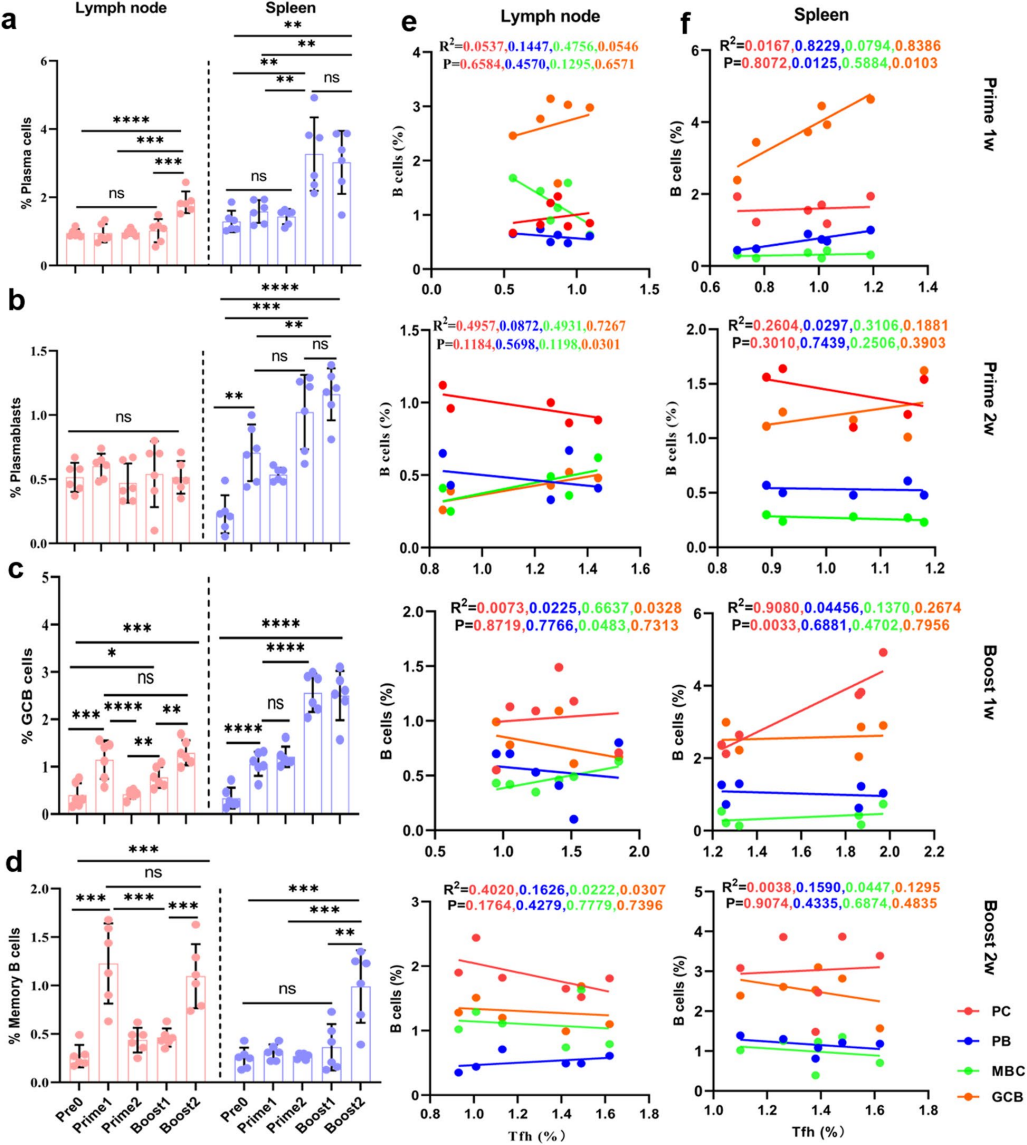
RBD protein-induced Tfh cell responses correlated with B cell responses. **a**-**d** Proportions of PC (**a**), PB (**b**) GC B cells **(c)** MBC (**d**) in lymph node and spleen after prime and booster vaccination. **e**, **f** Tfh cells correlated with PC, PB, GC B cells, and MBC in lymph node (**e**) and spleen (**f**) after prime and booster vaccination. Kruskal–Wallis’s test and Pearson correlation coefficient, n = 6. *: *P* < 0.05, **: *P* < 0.01, ***:* P* < 0.001, ****:* P* < 0.0001, ns: no significance

We wondered whether the vaccine-induced Tfh cells correlate with B cell responses. Firstly, we examined different B cell subsets including plasmablasts (PB), PC, GC B cells, and MBC after vaccination (Fig. S2) [24]. In spleen, we saw modest increased frequency of PBs and GC B cells after prime vaccination (*p* = 0.02, *p* < 0.0001, Fig. 4b, c). Booster vaccination led to a significant increase in their frequencies and numbers of PC and MBC (*p* = 0.002, *p* < 0.001, Fig. 4a-d). In lymph node, a significant augmented percentage of GC B and MBC was observed at day 7 after prime vaccination (*p* < 0.001, Fig. 4c, d). The frequency of PC, GC B, and MBC was significantly increased (*p* < 0.001, Fig. 4a-d) after booster vaccination.

Next Pearson Correlation Coefficient analysis was performed to analyze the correlation between Tfh cells and B cells. We noticed a positive correlation between Tfh cells and GC B cells in lymph node at day 14 after prime vaccination (R^2^ = 0.7267, *p* = 0.0301, Fig. 4e). The frequency of Tfh cells in spleen correlated positively with PB and GC B in spleen at day 7 after prime vaccination (R^2^ = 0.8229, 0.8386, *p* = 0.0125, 0.0103, Fig. 4f). Furthermore, there is a positive correlation between Tfh cells and MBC in lymph node (R^2^ = 0.6637, *p* = 0.0483, Fig. 4e) and between Tfh cells and PC in spleen at day 7 after booster vaccination (R^2^ = 0.9080, *p* = 0.0033, Fig. 4f). Collectively, these observations suggested RBD protein-induced Tfh cell responses correlated with B cell responses.

### IL-21 secretion by RBD-induced Tfh cells was vital to anti-RBD antibodies and B cells responses

**Fig. 5:**
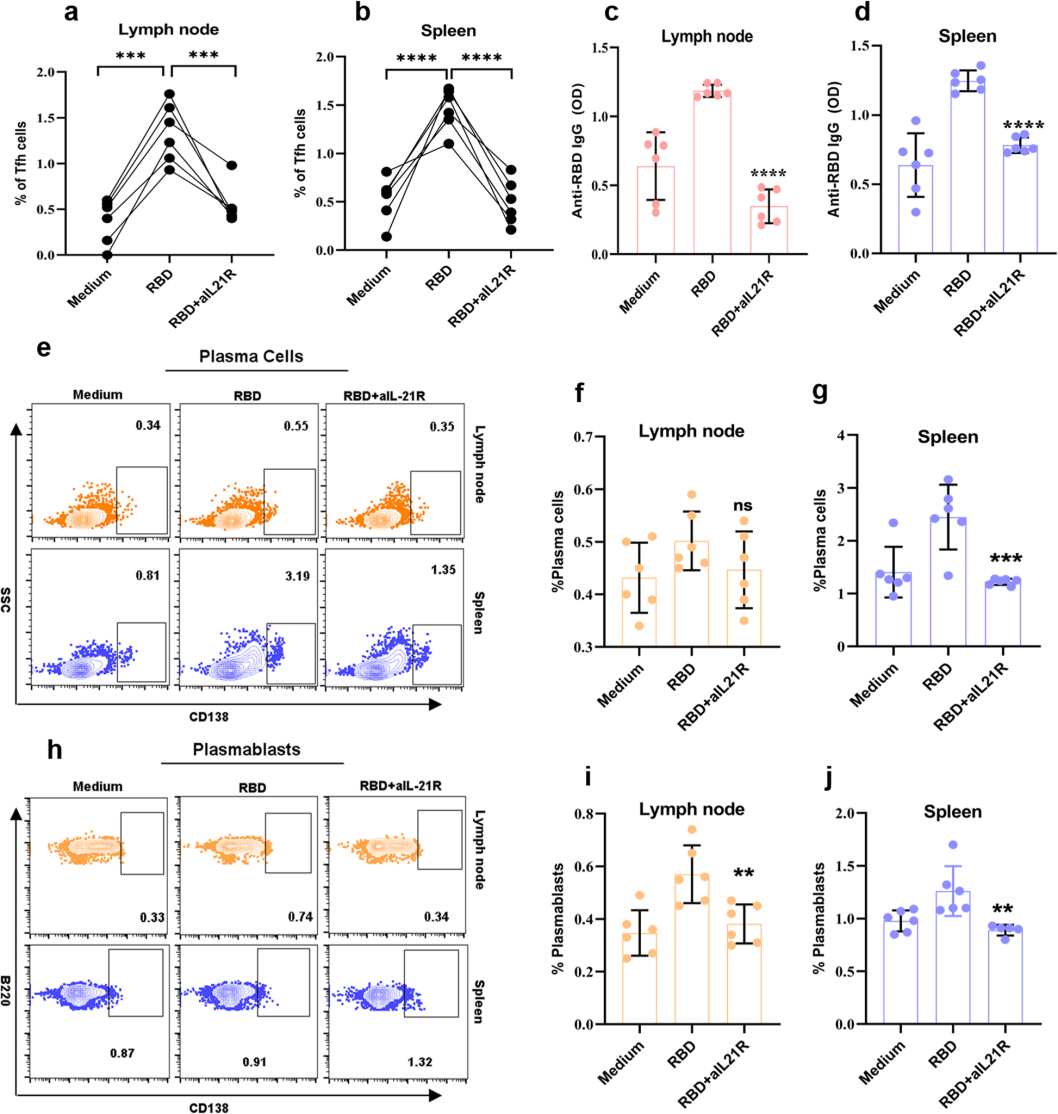
IL-21 secretion by RBD-activated Tfh cells was critical for anti-RBD antibodies and B cells responses. **a**-**b** Proportions of Tfh cells in lymph node (**a**) and spleen (**b**) following IL-21R blocking by adding IL-21R-Fc. **c**, **d** The concentrations of IgGs in the cultural supernatant from lymph node (**c**) and spleen (**d**) following IL-21R blocking by adding IL-21R-Fc. **e**, **h** Representative flow cytometry plots showing PC (**e**) and PB (**h**) percentage in lymph node and spleen following IL-21R blocking. **f**, **g** Proportions of PC in lymph node (**f**) and spleen (**g**). **i**, **j** Proportions of PB in lymph node **(i)** and spleen **j**. One way ANOVA test, n = 6. *: *P* < 0.05, **: *P* < 0.01, ***:* P* < 0.001, ****:* P* < 0.0001, ns: no significance

To determine whether the differentiation of B cells depends on IL-21 produced by Tfh cells, we blocked IL-21R by addition of IL-21R-Fc in the culture of lymphocytes from spleen and lymph nodes (Fig. 5). We noticed blocking IL-21 led to a significant inhibition of Tfh cells differentiation (*p* < 0.001, Fig. 5a, b). The ELISA results showed that blocking the expression of IL-21 resulted in a significant decrease in IgG secretion both in the spleen and lymph nodes (*p* < 0.001, Fig. 5c, d). Furthermore, we analyzed the frequencies of PB and PC in spleen and lymph nodes (Fig. 5e-j). In the lymph nodes, there was no change in PC (*p* > 0.05, Fig. 5f), however, we found a reduction of PB (*p* = 0.006, Fig. 5i). Blocking IL-21 resulted in a significant inhibition of PB and PC in spleen (*p* = 0.008, *p* = 0.0007, Fig. 5g, j). The help of Tfh cells to humoral response was a great extent rely on IL-21, as blocking IL-21 strongly inhibited antibodies production and B cells differentiation.

### CXCR5^+^CD4^+^T cells induced the differentiation of MBC towards PB and PC through IL-21 secretion

**Fig. 6:**
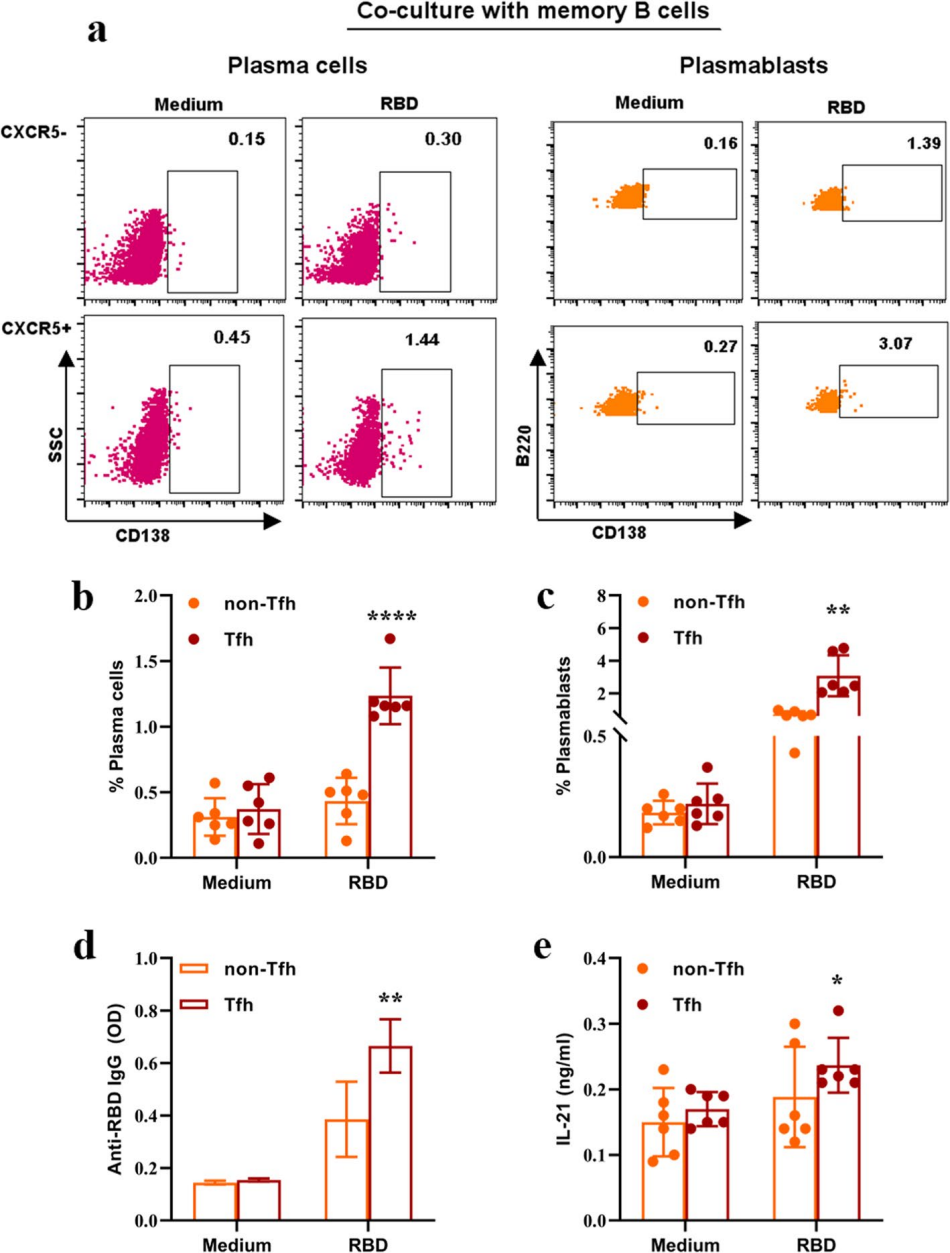
CXCR5^+^CD4.^+^T cells induced the differentiation of MBC towards PB and PC through IL-21. **a** Representative flow cytometry plots showing PC (left) and PB (right) after co-culturing with MBC. **b**, **c** Proportions of PC (**b**) and PB **c**. **d** ELISA detected the IgG antibody from cultural supernatant. **e** ELISA detected the IL-21 level from cultural supernatant. Kruskal–Wallis’s test, n = 6. *: *P* < 0.05, **: *P* < 0.01, ****:* P* < 0.0001

To verify the function of Tfh cells in B cell immune production, we sorted CXCR5^+^CD4^+^T cells and CXCR5^−^CD4^+^T cells from spleen after immunization and cultured with MBC from spleen, respectively. CXCR5^−^CD4^+^T cells failed to induce MBC to convert to antibody-secreting cells (Fig. 6a-c). In contrast, CXCR5^+^CD4^+^T cells efficiently fostered MBC to convert into antibody-secreting cells (*p* < 0.0001, *p* = 0.001, Fig. 6b, c). In addition, CXCR5^+^CD4^+^T cell group showed higher levels of IgG and IL-21 (*p* = 0.003, *p* = 0.02, Fig. 6d, e). However, no significant difference was observed in CXCR5^−^CD4^+^T cells, which demonstrated that CXCR5^+^CD4^+^T cells produced IL-21 upon contact with MBC, whereas CXCR5^−^CD4^+^T cells scarcely produced IL-21.

## Discussion

Efficacious vaccines provide protection against pathogens by inducing longevous PC and MBC to produce long-lasting high-affinity antibodies [25]. Such humoral immunity needs the interaction of Tfh cells and B cells [25]. Loss of function of Tfh cells can lead to impaired humoral immunity and aggravate the severity of the disease [26]. Recent studies showed an increased circulating Tfh cells (cTfh) frequency in COVID-19 patients was accompanied by elevated antibodies levels [26, 27]. In our study, the recombinant SARS-CoV-2 RBD protein expressed in *Drosophila* S2 cells elicited remarkable antigen-specific Tfh cell reactions in lymph node and spleen. Furthermore, ICOS^+^CXCR5^+^CD4^+^T cells recognized SARS-CoV-2 RBD antigen at day 7 following vaccination. This observation might hint that the activation process of functional Tfh cells initiated immediately after vaccination. Booster vaccination led to a robust increase in CXCR3^+^ (Tfh1 and Tfh1-17) subset and modestly increased frequency of CXCR3^−^ (Tfh2 and Tfh17) subset in spleen and lymph node. The response of CXCR3^+^ (Tfh1 and Tfh1-17) subset was stronger than that of CXCR3^−^ (Tfh2 and Tfh17) subset. The Th1‐polarizing conditions of a viral infection usually result in the predominant generation of type‐1 Tfh cells, such as in SARD-CoV-2 infection [28], influenza vaccination [7, 29], live‐attenuated yellow fever vaccination [30], and Hepatitis C Virus infection [31]. A nonadjuvanted trivalent split seasonal influenza vaccine up-regulated the frequency of CXCR3^+^ cell [32]. The Tfh17 cell subset was induced during the antibody response to rVSV-ZEBOV Ebola vaccine [33]. The Tfh1 subset dominated in response to influenza vaccine [7, 33]. A study on SARS-CoV-2 mRNA-LNP vaccines showed Tfh1 and Tfh17 responses were detected in human vaccinated with mRNA-LNP vaccines [10]. *Mingjuan *et al. reported that a skewed cTfh population away from cTfh2 and cTfh17 toward cTfh1 in the low responders to hepatitis B vaccine [5].

Focus was then toward studying the effect of the RBD protein on Tfh cell function. We found that RBD vaccination induced a predominant Th1 type response. Activated Tfh highly expressed PD-1, which interacted with PD-L1 on B cell surface to promote affinity maturation of B cells. IL-21 and Bcl-6 have been known as critical factors for Tfh cell development [34]. Our result showed the expression of PD-1 was significantly increased after vaccination especially at the early stage. The levels of IL-21 and Bcl-6 showed remarkable increases after vaccination, which supported the notion that RBD-induced functional Tfh cell participated in cellular responses against SARS-CoV-2. The result was consistent with previous study [24].

Due to the key role of Tfh cells in T cell-dependent antibody response, we further analyzed the relevance of Tfh cell with B cell. Notably, we detected GC B cell responses in spleen and lymph node at day 7 after prime vaccination, which indicated prime vaccination with RBD protein elicited the GC reactions in secondary lymphoid organs. More robust GC B cell responses were observed following booster vaccination. In addition, we found booster vaccination promoted different B subsets responses including PC, PB, and MBC in spleen and lymph node, which suggested that booster vaccination was necessary for long-lasting humoral immune memory responses. Importantly, there was a correlation between Tfh cells and GC B cells, PB, and PC in spleen, and between Tfh cells and MBC in lymph node at early stage after RBD vaccination. These observations implied that RBD-induced Tfh cells may provide help to antigen-experienced B cells at follicular sites and contribute to generate high-affinity antibodies against SARS-CoV-2. In fact, the notion that high-affinity antibody formation relies on Tfh cell response has been widely reported in influenza fields [6, 7].

IL-21 is a representative cytokine of Tfh cells and is essential for the function of Tfh cells [35]. Consistent with previous studies from influenza vaccines [6, 7], an IL-21 blocker demonstrated significant reductions of Tfh cells, PB, and PC frequencies in spleen and lymph node, which indicated that IL-21 was mainly secreted by Tfh cells, and the secretion of IL-21 by Tfh cells was essential for B cell differentiation. Furthermore, the production of IgG in the cultural supernatant in spleen and lymph node after IL-21 signaling blocking was diminished. This suggested that Tfh cells might be involved in the process of vaccine‐induced humoral response through IL‐21 secretion.

Studies on influenza vaccines demonstrated that vaccine mainly activated MBC to promote antibody responses [6, 36, 37]. Our study showed that RBD protein-induced Tfh cells but not non-Tfh cells efficiently promoted MBC to differentiate into antibody-secreting cells. The ELISA result showed that the levels of IgG and IL-21 in the cultural supernatant from Tfh and MBC co-culture stimulated with RBD protein were significant increased. This was an important finding that strengthened the notion that RBD protein-induced functional Tfh cells might promote antibody responses mainly by enhancing the memory response. This finding was similar to previous reports for papillomavirus vaccines and influenza vaccines [7, 25].

Our study has several limitations. The ideal experiment would be a combination of in *vivo* and in *vitro*, but we only performed the IL-21R blockade assay in *vitro*. However, *Abdullah *et al. and *Rimpei *et al. have demonstrated that IL-21 secreted by Tfh cells help B cells differentiation through the IL-21R blockade assay in *vitro* after influenza vaccine, and our result was consistent with previous studies [6, 38]. In addition, our study only measured spleen and lymph node; however, the function of cTfh cells in peripheral blood also need to be monitored.

In summary, our results demonstrated the induction of antigen-specific Tfh cells in secondary lymphoid organs after vaccination with recombinant RBD protein and showed their essential role in the vaccine-induced humoral immune response. The study suggested that inducing antigen-specific Tfh cell response after vaccination may provide an efficacious vaccination strategy and have important implications for designing next-generation vaccines.

## Material and method

### Expression and purification of SARS-CoV-2 RBD antigen

The DNA sequence encoding the SARS-CoV-2 S protein RBD (YP_009724390.1: Asn 331-Lys529) that was codon-optimized for expression in *Drosophila* S2 cells was synthesized, amplified by PCR and cloned into the pMT/BiP/TEV-His expression vector with a 6 × His tag for affinity purification. This vector was modified from the pMT/BiP/V5-His plasmid (Invitrogen) by replacing the V5 epitope with the tobacco etch virus (TEV) protease recognition site to enable specific removal of the C-terminal 6 × His tag of fusion proteins [19]. The construct was confirmed by sequencing.

Stably transfected S2 cells were generated by co-transfection of the construct and the pCoBlast selection vector encoding Blasticidin S deaminase at a ratio of 19:1 using Cellfectin II transfection reagent. Two days post transfection, S2 cells stably expressing RBD protein were selected by addition of 25 μg/ml Blasticidin S (Invovogen) to the SFX-Insect medium (Hyclone). The selection medium was replaced every 4 days and stable cell lines were established in ~ 3 weeks.

Stably transfected S2 cells were grown in shaker flasks to a density of 8 × 10^6^ cells/ml and were induced with 0.5 mM CuSO_4_ for protein expression. After induction, cells were grown in the SFX-insect medium for an additional 5 days [39]. The cells were harvested by centrifugation at 4000 rpm for 15 min followed by passing through a 0.22 μm filter to remove cell debris. The supernatant was applied to a HisTrap Excel 5 ml column (Cytiva) at 5 ml/min followed by 20 CV wash with 50 mM Tris, 500 mM NaCl, 1 mM imidazole, pH 8.0. Bound proteins were eluted with 50 mM Tris, 500 mM NaCl, 500 mM imidazole, pH 8.0. Protein was further purified using size exclusion chromatography (SEC) by loading pooled eluate onto a HiLoad 16/600 Superdex 200 pg column (Cytiva) equilibrated in PBS buffer. Fractions containing the protein of interest were pooled, concentrated in 10 kDa MWCO concentrator (Amicon Ultra), aliquoted, and frozen at -80 °C. Protein concentration was determined using Nanodrop 2000 (Thermo Fisher). The quality of protein was assessed by sodium dodecyl sulfate–polyacrylamide gel electrophoresis (SDS-PAGE).

### Animal immunization

Six- to 8-week-old female C57BL/6 mice were purchased from the Animal Laboratory Center of Ningxia Medical University. All animal experiments followed the Ningxia Medical University Institutional Review Committee with approved number 2022-Z052. Mice were randomly classified into two groups, immune and control groups. The recombinant RBD protein in alum was prepared by mixing RBD protein in PBS with.

Alum (Thermo Fisher Scientific, 77,161) at a 1:1 ratio. The immune group was immunized with 100 µL of solution containing 20 µg SARS-CoV-2 RBD protein by abdominal subcutaneous in a 2-week interval. Primary vaccination was done 3 times [24]. Then, some mice were sacrificed at day 7 and 14 following final prime vaccination. The remaining mice were rested for 4 weeks, then, the immune group was given 2 booster injections of RBD protein/ alum mixture in a 2-week interval [40]. Mice were then sacrificed at day 7 and 14 following final booster vaccination. All control group was injected with PBS.

### In vitro cell culture

*Blocking IL-21R assay*: 5 × 10^6^ cells from spleen and lymph node were co-incubated with 10 µg/mL recombinant mouse IL-21R Fc chimera (Bio-Techne, 991-R2-100) for 1 h prior to stimulation by RBD protein. After 48 h, cells were harvested and detected by flow cytometry and culture supernatants were harvested and detected by ELISA.

*T cells and B cells co-culture*: Sorted CD4^+^CXCR5^+^ and CD4^+^CXCR5^−^ cells (5 × 10^4^ cells per well) were cocultured with MBC (5 × 10^4^ cells per well), respectively, in the presence of 10 µg/mL recombinant RBD protein and 1 µg/mL endotoxin-reduced SEB (Sigma) for 6 days in 96-well plates.

### Flow cytometry

All FCM antibodies were purchased from Biolegend. Cells were resuspended in Buffer 2 (as previous reported [41]). The following antibodies were used to analyze T cells and intracellular cytokine staining: CD4-APC/Cyanine7, CXCR5-Brilliant Violet 421, PD-1-PerCP/Cyanine5.5, CD3-Alexa Fluor700, ICOS-FITC, CXCR3-PE, CD154-APC, IL-21-PE. The following antibodies were used to analyze B cells: CD45-PE, B220-FITC, IgD-Brilliant Violet 510, GL7-APC, CD138-Brilliant Violet 421, CD38-Alexa Fluor 700, and Fas-BV605.

### ELISpot analysis

Mouse IFN-γ and IL-4 ELISpot analysis was performed according to product manuscripts (Mabtech). In brief, lymphocytes from spleen were cultured in 48-well plate at 1 × 10^6^ cells/well in the present of 10 μg/mL RBD protein for 24 h. Then the plates were washed 4 times with sterile PBS (200 μL/well) and closed with 1640 medium containing 10% serum incubated for 1 h at room temperature. The medium was removed and the cells were added into the plate. The plate was putted into the incubator for 48 h and then washed 5 times with PBS. Next, 100 μL 1 μg/ml detection antibody was added into well and incubated for 2 h at 37℃, and the plate was washed as above. Streptavidin-ALP was diluted in PBS-0.5% FCS and added 100 μL into each well, and incubated 1 h at 37℃. 100 μL substrate solution was added in plate for color developed and double distilled water was added to stop reaction. Dry plate was read through AID ELISpot Reader Classic.

### ELISA for detecting cytokines

IL-21 was detected through mouse IL-21 ELSA kit (Solarbio) according to the manufacturer’s protocol. In brief, wash the plate 3 times and shake dry before adding the standard/sample. Add 100 µL standard and test samples to the reaction wells, and incubate in 37 ℃ incubator for 90 min. Wash the plate 4 times. Then add in 100 µL biotin antibody working fluid to the reaction wells, and incubate in 37 ℃ incubator for 60 min. Wash the plate 4 times. Add in 100 µL enzyme combination working liquid to the reaction wells, and incubated in 37 ℃ incubator for 30 min. Wash the plate 5 times. Add 100 µL chromogenic substrate to the reaction wells, and plates were developed for 15 min. Finally, add 50 µL stop solution to stop reaction. The reaction plates were read at 450 nm.

### Real time RT-PCR

Total RNA was extracted from splenocytes using TRIzol reagent (Invitrogen). Real-time PCR was set up with Revert Aid First Strand cDNA Synthesis Kit (Thermo Fisher Scientific, K16215) and Bestar™ qPCR MasterMix (SYBR Green) (DBI Bioscience). qRT-PCR for Bcl6 was performed using the following primer pairs: Bcl-6: Forward 5’- CCTGTGAAATCTGTGGCACTCG and reverse 5’- CGCAGTTGGCTTTTGTGACG; and GAPDH: Forward 5’-AGGTCGGTGTGAACGGATTTG and reverse 5’-GGGGTCGTTGATGGCAACA.

### Statistical analysis

Using Graphpad prism 8 and Origin 2021 to analyze the experimental results. Comparing the differences between two groups was evaluated by two-tailed Student’s t test. One way ANOVA and Kruskal–Wallis were conducted to evaluate multiple comparisons. Correlation was assessed by Pearson correlation coefficient. *P* values of < 0.05 were considered significant.

### Supplementary Information

**Additional file 1: Supplementary Fig. 1.** Representative gating strategy for Tfh cells. Representative flow cytometry plots showing Tfh cells after vaccination. **Supplementary Fig. 2.** Representative gating strategy for different B cell subsets. Representative flow cytometry plots showing PB, PC, MBC, and GC B cells after vaccination.

## Data Availability

This study-related data can be obtained from the corresponding author.
