# Correction: Activation and induction of antigen-specific T follicular helper cells play a critical role in recombinant SARS-CoV-2 RBD vaccine-induced humoral responses

**DOI:** 10.1186/s43556-023-00152-0

**Published:** 2023-12-18

**Authors:** Songhao Yang, Liangwei Duan, Chan Wang, Cuiying Zhang, Siyu Hou, Hao Wang, Jiahui Song, Tingting Zhang, Zihua Li, Mingxia Wang, Jing Tang, Qianqian Zheng, Hui Wang, Qi Wang, Wei Zhao

**Affiliations:** 1 School of Basic Medical Science, Ningxia Medical University, Yinchuan, Ningxia Hui Autonomous Region 750004 People’s Republic of China; 2 Key Laboratory of Hydatid Disease, Ningxia Medical University, Yinchuan, Ningxia Hui Autonomous Region 750004 People’s Republic of China; 3 Center of Scientific Technology, Ningxia Medical University, Yinchuan, Ningxia Hui Autonomous Region 750004 People’s Republic of China; 4 Henan Key Laboratory of Immunology and Targeted Drugs, School of Laboratory Medicine, Xinxiang Medical University, Xinxiang, 453003 Henan Province China; 5 Henan Collaborative Innovation Center of Molecular Diagnosis and Laboratory Medicine, Xinxiang Medical University, Xinxiang, 453003 Henan Province China

**Correction: Mol Biomed 4, 34 (2023)**

**https://doi.org/10.1186/s43556-023-00145-z**

Following publication of the original article [1], the authors reported that a funding number needed to be added in the funding section.

The original version is:

This research was supported by the Open project of the Key Laboratory of Hydatid Disease of Ningxia Medical University, the Key Scientific and Technological Project of Henan Province (Grant No. 222102310025), the Project for Young Scientists of Henan Province, the International Joint Research Laboratory for Recombinant Pharmaceutical Protein Expression System of Henan (Grant No. KFKTYB202210), and the 111 Project (Grant No. D20036).

The updated version is:

This research was supported by the Open project of the Key Laboratory of Hydatid Disease of Ningxia Medical University, the Key Scientific and Technological Project of Henan Province (Grant No. 222102310025), the Project for Young Scientists of Henan Province (Grant No. 225200810074), the International Joint Research Laboratory for Recombinant Pharmaceutical Protein Expression System of Henan (Grant No. KFKTYB202210), and the 111 Project (Grant No. D20036).

The original article [1] has been corrected.

## References

[1] Yang S, Duan L, Wang C (2023). Activation and induction of antigen-specific T follicular helper cells play a critical role in recombinant SARS-CoV-2 RBD vaccine-induced humoral responses. Mol Biomed.

