# Supplementary material for: Activation and induction of antigen-specific T follicular helper cells play a critical role in recombinant SARS-CoV-2 RBD vaccine-induced humoral responses
Source: Mol Biomed. 2023 Oct 19;4:34. doi: 10.1186/s43556-023-00145-z (PMC10584785; doi:10.1186/s43556-023-00145-z)

**Activation and induction of antigen-specific T follicular helper cells play a critical role in recombinant SARS-CoV-2 RBD vaccine-induced humoral responses**

Songhao Yang^1,2,3#^, Liangwei Duan^4,5#^, Chan Wang^1,2#^, Cuiying Zhang^1,2^, Siyu Hou^1,2^, Hao Wang^4,5^, Jiahui Song^2,3^, Tingting Zhang^1,2^, Zihua Li^1,2^, Mingxia Wang^1,2^, Jing Tang^1,2^, Qianqian Zheng^4,5^, Hui Wang^4,5^, Qi Wang^1*^ and Wei Zhao^1,2,3*^

1 School of Basic Medical Science of Ningxia Medical University, Yinchuan, Ningxia Hui Autonomous Region 750004, PR China.

2 Key Laboratory of Hydatid Disease of Ningxia Medical University, Yinchuan, Ningxia Hui Autonomous Region 750004, PR China;

3 Center of Scientific Technology of Ningxia Medical University, Yinchuan, Ningxia Hui Autonomous Region 750004, PR China;

4 Henan Key Laboratory of immunology and targeted drugs, School of Laboratory Medicine, Xinxiang Medical University, Xinxiang 453003, Henan Province, China.

5 Henan Collaborative Innovation Center of Molecular Diagnosis and Laboratory Medicine, Xinxiang Medical University, Xinxiang 453003, Henan Province, China.

* Corresponding author: Qi Wang, E-mail, [wqmam@126.com](mailto:wqmam@126.com). Wei Zhao, E-mail, Weizhao@nxmu.edu.cn.

# First Author: Songhao Yang, Liangwei Duan and Chan Wang contributed equally to this work.

**Supplementary Fig. 1 Representative gating strategy for Tfh cells**

**
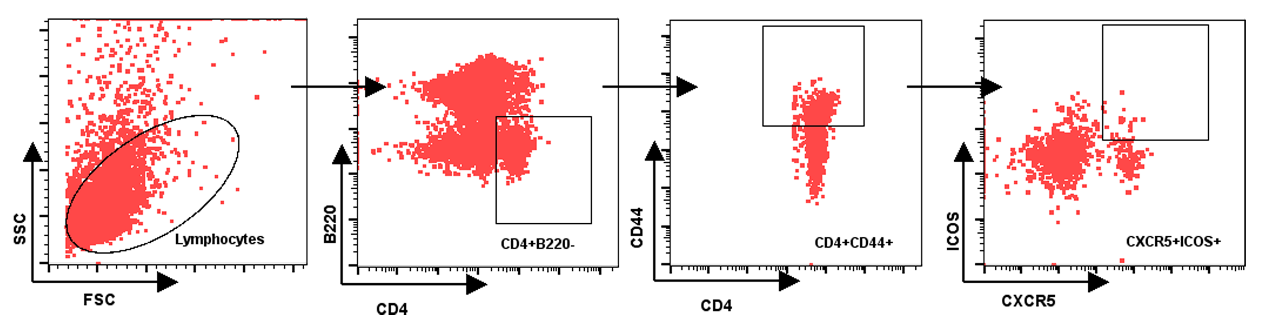
**Representative flow cytometry plots showing Tfh cells after vaccination.

**Supplementary Fig. 2** **Representative gating strategy for different B cell subsets** Representative flow cytometry plots showing PB, PC, MBC, and GC B cells after vaccination.


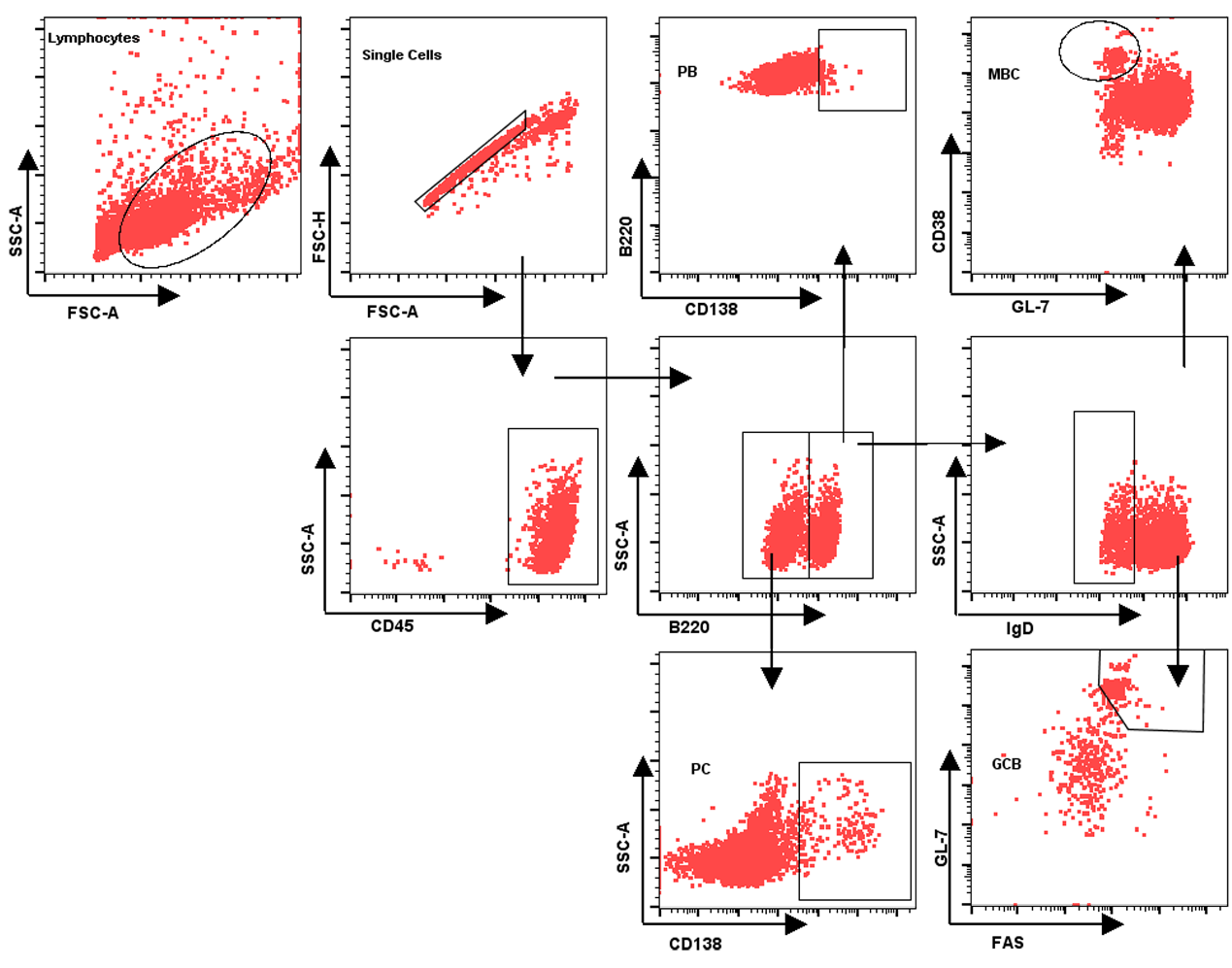

Supplement: Supplementary file 1 — Additional file 1: Supplementary Fig. 1. Representative gating strategy for Tfh cells. Representative flow cytometry plots showing Tfh cells after vaccination. Supplementary Fig. 2. Representative gating strategy for different B cell subsets. Representative flow cytometry plots showing PB, PC, MBC, and GC B cells after vaccination. [file 43556_2023_145_MOESM1_ESM.docx]
